# How to … do mixed‐methods research

**DOI:** 10.1111/tct.13145

**Published:** 2020-02-24

**Authors:** Anu Kajamaa, Karen Mattick, Anne de la Croix

**Affiliations:** ^1^ Faculty of Educational Sciences University of Helsinki Helsinki Finland; ^2^ Centre for Research in Professional Learning University of Exeter Exeter UK; ^3^ LEARN! Academy Vrije Universiteit Amsterdam Amsterdam the Netherlands; ^4^ Research in Education, Amsterdam UMC VUmc School of Medical Sciences Amsterdam the Netherlands

## Abstract

As a clinician, you will often combine patients’ narratives with test results in order to obtain a coherent picture and then decide on a way forward. As an educator, you are also likely to combine different information from your learners to arrive at the best feedback, judgement or supervision plan. This is what researchers do when undertaking mixed‐methods research: qualitative and quantitative data are typically brought together to provide different insights than could be achieved with a single type of data and analysis. Mixed‐methods research has much to offer the clinical teacher but may involve more complex study designs than other types of research. Therefore, this article aims to highlight the different designs of mixed‐methods research, and the opportunities and challenges that it provides, in order to support researchers who may be undertaking their first mixed‐methods research study.

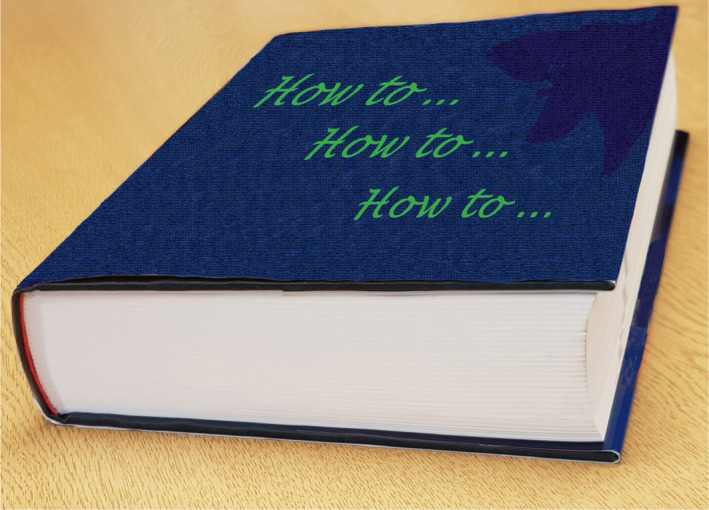

## What is mixed‐methods research?

Mixed‐methods research, or multi‐strategy designs,^1^ can be defined as ‘the collection, analysis and integration of both qualitative and quantitative data in a single study’:^2^ semi‐structured interviews and workplace measures (e.g. attendance data) might be undertaken concurrently to gain a multifaceted perspective on a particular phenomenon; a survey with closed‐answer questions might be followed by semi‐structured interviews in order to gain an in‐depth understanding of its key findings; or interviews can be used to develop a survey. The term multiple methods is sometimes applied when more than one qualitative (or quantitative) method is used within a single study. It can also be used to describe the combination of more than one qualitative or quantitative method within the same research study.^1^ Definitions for ‘mixed‐methods research’ are not static or universally agreed, however, as the field is relatively young and is constantly developing.^3^ Some have argued that the term ‘mixed methods’ is misleading, as it suggests that the element of mixing sits at the level of the choice of methods only, whereas the integration of qualitative and quantitative research components should, in fact, be considered at the level of the whole research process. When used as an overarching lens to guide the formulation of aims for the research, the research gap or questions, the research design, and the data collection, data analysis and reporting of the results, however, mixed‐methods research can expand and strengthen the conclusions and contributions of a study.^4^

… mixed‐methods research can expand and strengthen the conclusions and contributions of a study

## What designs of mixed‐methods research are there?

Before designing the study it is important to describe the phenomenon under study, and the aim or the research question to be used for the study, in order to explain why mixed‐methods research is needed. This enables the researcher to link the philosophical underpinning of the study to its research design.^5^ The role of the qualitative data can be to supplement a predominantly quantitative design: for example, a qualitative process evaluation alongside a clinical trial. Similarly, the role of the quantitative data can be to supplement a predominantly qualitative design: for example, the use of a quantitative measure to provide further insights to enrich the qualitative findings within an exploratory case study. Or, if you rely on pragmatism,^6^, ^7^ which is a commonly applied paradigm in mixed‐methods research, you are likely to orient the study design towards solving practical problems in the ‘real world’.^8^

There are many types of mixed‐methods research design. These include: (i) sequential exploratory mixed‐methods design, which can be characterised by initial qualitative data collection and analysis, followed by a phase of quantitative data collection and analysis, leading to the integration or linking of data from the two separate corpuses of data to further explore, develop and test the qualitative analysis; (ii) sequential explanatory mixed‐methods design, which usually implies collecting, analysing and connecting quantitative and then qualitative data in two consecutive phases, resulting in integrating the findings within one study, in order to explain quantitative results using qualitative findings; (iii) convergent mixed‐methods study design with quantitative and qualitative data that aim to identify converging evidence that corroborates the validity of the conclusions drawn from different methods and data sources; and (iv) nested mixed‐methods study, in which qualitative and quantitative components sit alongside one another, but with one component clearly dominant and the other nested or embedded within it, to improve the quality of the conclusions. In Table 1, we provide four exemplar studies that have been selected to illustrate the mixed‐methods designs described here.^9^, ^10^, ^11^, ^12^

**Table 1:** Examples of mixed‐methods research studies

Type of mixed‐methods study design	Example
Sequential exploratory	Fisher and colleagues designed a mixed‐methods study to explore the prescribing activities of hospital pharmacists.^9^ The study had a sequential exploratory design: first, in a qualitative phase, 27 people were interviewed individually or in a focus group and the data were analysed, with the results grouped into themes. Then, in the quantitative phase, a cross‐sectional survey (*n* = 274) was designed, using the themes resulting from the qualitative data analysis to create the items. Integration was achieved in the design, by having the second part of the study build upon the findings of the first part. The results in the study are reported contiguously: i.e. first the qualitative findings, then the quantitative findings
Sequential explanatory	Shahhosseini and Hamzehgardeshi studied nurses’ perceptions of common facilitators and barriers to participation in continuing education programmes.^10^ To do so, they also used a sequential approach, but they started with the quantitative phase, using questionnaires (*n* = 361). In the second, qualitative phase, they made use of interviews. They interviewed 25 nurses to ask them about their perceptions and analysed the interviews using content analysis. Integration in this study mainly took place at the level of interpretation and reporting: it is in the discussion that both analyses are combined. The data sets of both sub‐studies are connected, as the interviewees were sampled from the wider data set of the quantitative phase. It is unclear whether the findings from phase 1 were used to inform phase 2 (e.g. sampling, question construction). The results are also presented contiguously
Convergent	Rosenkranz and colleagues made use of a convergent design to understand (de)motivating factors for medical students to do research.^11^ The qualitative and quantitative part were undertaken in parallel, by a cross‐sectional survey (*n* = 579) and interviews (*n* = 23). The data sets were both analysed with the same theoretical framework (Self‐Determination Theory). Equal weight was given to both the qualitative and quantitative elements of the study. After analysis, integration took place in the development of a model, at the level of interpretation and reporting. The results are presented in a weaved manner, i.e. with themes illustrated by both sets of data. By using one theoretical framework, the authors made a strong joint display of findings to achieve integration
Nested	Grocke and colleagues wanted to know whether people with severe mental illness could benefit from music therapy.^12^ To do so, they undertook a randomised controlled trial, with a cross‐over design. The intervention was singing songs, composing and recording songs, and they measured the effect on quality of life via questionnaires. The quantitative study was the main focus, yet two qualitative elements were nested, or embedded, in the study. Focus group interviews were undertaken after the intervention and song lyrics of self‐composed songs were analysed. Qualitative themes were embedded within the quantitative outcomes to provide a better understanding of the intervention than either approach alone. There was a clear connection between the sub‐studies, as they dealt with the same study population. The integration took place mainly in the interpretation and reporting phase, as both data sets were analysed separately. The results are presented in a contiguous way

Whatever mixed‐methods research design is used, achieving the highest quality lies in describing what was done (why, how and when) in detail, and being reflexive.^13^ In other words, it is important to be sensitive to the limitations of the study and to report this openly and honestly, referring to methodological references such as those cited by this article. Table 2 provides a tool that we hope will help novice researchers justify their need for more than one type of method to address their study aim and to navigate the key choices available to them. The table is based on several existing frameworks and designs of mixed‐methods research,^1^, ^4^ which introduce key terminology and concepts to guide the research process.

**Table 2:** Questions to be asked in the design of mixed‐methods research studies

Questions	Explanation and prompts
What is the *overarching aim* of the study?	Mixed‐methods studies, by definition, are often designed with a specific aim that can guide the final study design: discuss with the research team whether the overarching aim is theory building (explaining, exploring or describing phenomena) or hypothesis testing
Which is the *dominant method*?	In some mixed‐methods studies the methods are equally weighted but often they are not. It is worth making this explicit. Nested or embedded designs refer to where there is a smaller data set collected within a larger study for a specific purpose
Is the data collection *sequential*,* in parallel* or *convergent*?	Research designs may be described as sequential (one after the other), in parallel (happening concurrently but separately, with integration occurring later) or convergent (happening concurrently and with the data sets interacting)
At what stage does the *integration* of the two methods occur?	It is important to be clear about whether, when, to what extent and how integration was achieved in the methodology section of the study
Is the qualitative element *explanatory* or *exploratory*?	The qualitative element of the mixed‐methods research may have a range of different purposes, such as explaining previous findings or exploring a phenomenon

## What are the opportunities provided by mixed‐methods research?

Mixed‐methods research is well placed to investigate complex phenomena and situations, and can provide researchers with more nuanced understanding of certain phenomena than the use of single methods.^14^ Mixed‐methods research can provide a powerful tool for investigating complex processes and systems in health and social care that draw upon the strengths of both quantitative and qualitative approaches.^4^, ^8^ Therefore, mixed‐methods research enables the researcher to answer different kinds of research questions than the questions that could be answered by qualitative or quantitative methodologies alone.

Mixed‐methods research may be useful when researchers want to consider an activity from different levels of a system (e.g. individual, ward, department, hospital and health care system), when they want to compare results from different sources or when they want to illustrate trends or consider processes alongside outcomes.^8^ It can also be useful to undertake a quantitative study to locate or define an appropriate sample for qualitative research, and to explore contradictions in existing workplace measures or reports.^5^ The qualitative and quantitative methods must both be sound for the mixed‐methods study to be of high quality, as one method cannot compensate for the other. To ensure rigorous research conduct quality appraisal tools are available for mixed‐methods studies, such as the Mixed Methods Appraisal Tool.^15^

Lastly, mixed‐methods research findings may ‘speak’ to diverse audiences, with those who are persuaded by statistics being persuaded by the quantitative element of the study, and with those who are persuaded by user experiences and stories being more persuaded by the qualitative element.

… mixed‐methods research findings may ‘speak’ to diverse audiences …

## What are the challenges with mixed‐methods research?

When mixing qualitative and quantitative methods, it is critically important to justify why, how and when qualitative and quantitative methods are being combined.^1^ It can be more challenging for mixed‐methods research studies than single‐methods research studies to demonstrate clear alignment, from the research aim to the conclusion. When reporting mixed‐methods research in journals, it is often quite challenging to provide full transparency in describing the level of integration of the data, the various individual methods and the findings within a study.^14^

… it is critically important to justify why, how and when qualitative and quantitative methods are being combined

Mixed methods may involve multiple team members and/or advisors, who may well have different world views and ways of working, given their different methodological preferences. If different people are responsible for different parts of the study, collaborative efforts will be needed from the full team to contribute to the study design and to the research process, to ensure that true integration is achieved.^8^ In other words, diversity in viewpoints is likely to benefit mixed‐methods research but requires an investment of time. For these reasons, mixed‐methods research is typically quite resource intensive. We recommend that novice researchers collaborate with more experienced colleagues and protect time to read key references and access other resources that can help them. Moreover, interdisciplinary research is now being recommended and is becoming more common, but these different ways of working need to be considered early on in the design of a research project.

Optimising the integration of the study design, data collection, data interpretation and reporting is another challenge for mixed‐methods research.^16^, ^17^ For example, Bryman reported a lack of integration of the qualitative and quantitative components within mixed‐methods research articles, highlighting the need for researchers to focus on this aspect.^16^ Without appropriate research skills for managing the (often) multiple points of integration, the overall research study can seem unfocused or disjointed. Fetters and colleagues describe the challenges and potential solutions for integration in more detail than we are able to include here.^4^ It is also important for researchers using mixed methods to take into consideration ethical issues associated with qualitative and quantitative research procedures, such as the protection of the anonymity of participants.

## Conclusions

In this introduction to mixed‐methods research, we have provided a brief glimpse into different types of mixed‐methods research and their opportunities and challenges. We believe the potential of mixed‐methods research is great and particularly well suited to the kinds of complexity inherent in health care and education environments, where both qualitative and quantitative measures are needed to improve practice. To get started with a mixed‐methods research study, we advocate working through the questions posed in Table 2, namely: What is the overarching aim of the proposed study?; Why are mixed methods needed to address it?; and How and when will the data be integrated? We believe that exploring these questions will help to ensure the high quality of future mixed‐methods research.
